# Developmental and Reproductive Biology of the Ectoparasitoid, *Elasmus steffani*, in a Substitute Host, *Ephestia kuehniella*

**DOI:** 10.1673/031.010.11901

**Published:** 2010-07-30

**Authors:** I. Redolfi, M. Campos

**Affiliations:** ^1^ Universidad Nacional Agraria La Molina, Departamento de Biología, Ap. Postal 456, Lima, Perú; ^2^ Estación Experimental del Zaidín (CSIC), Department of Environmental Protection, Profesor Albareda I, 18008- Granada, España

**Keywords:** oviposition, feeding, longevity, olive orchards, *Prays oleae*

## Abstract

*Elasmus steffani* (Viggiani) (Hymenoptera: Elasmidae) is a gregarious idiobiont ectoparasitoid of *Prays oleae* (Bernard) (Lepidoptera: Plutellidae), an olive crop pest. In the substitute host *Ephestia kuehniella* (Zeller) (Lepidoptera: Pyralidae), the duration of the developmental stages was approximately 11–15 days. The preoviposition was 8.9 ± 5.0 days, and oviposition lasted 30.4 ± 10.5 days, with a reproduction capacity of 185.5 ± 62.3 eggs per female, for a mean of 5.4 ± 0.9 eggs per day. The oviposition rhythm reached its maximum when the parasitoid was 35 days of age. The lack of food negatively influenced survival of the adults, while those fed on a honey-water mixture lived significantly longer that those that also had access to a host as food. The female parasitoid fed upon 15% of the paralyzed larvae. The virgin female *E. steffani* exhibits arrhenotokic parthenogenesis.

## Introduction

The disadvantages of pesticide application in olive orchards encourage pest control to be oriented toward integrated management (Viggiani 1989; Katsoyannos 1992; Civantos and Sánchez 1993; Longo 1995; Hilal and Ouguas 2005), where biological control plays an important role (Jiménez 1984; Jervis et al. 1992; Delrio 2007; Hegazi et al. 2007), given that this agricultural ecosystem is rich in auxiliary insect fauna (Arambourg 1986; De Andrés 1991; Blibech et al. 2005). However, the development of biological control requires a thorough knowledge of the entomophagous insects that could be used as control agents. A candidate genus is *Elasmus*, species of which parasitize different phytophagous insects that constitute major pests of the olive, such as *Zeuzera pyrina, Euphyllura olivina*, and especially the last stages of the larvae *Prays oleae*, which is a major pest of the olive throughout most of the olive-growing zones of the Mediterranean Basin (Campos 1976; Arambourg 1986; Civantos 1999).

The species *Elasmus steffani* (Viggiani) (Hymenoptera: Elasmidae), is a gregarious idiobiont ectoparasite, and the females oviposit in the cocoon containing the last host larval stage. Different aspects of its biology have previously been studied (Campos 1981). In some zones, it is ranked as the most important regulator of the phyllophagous generation, and the second most important in the anthophagous generation of *P. oleae*, in some years reaching high parasitism rates (Campos and Ramos 1981; Katsoyannos 1992; Bento et al. 1998). This Elasmidae, usually associated with the olive (*Oleae europaea*) (Graham 1995; Torres 2007), has also been recorded within the complex of the most abundant parasitoids of *Lobesia botrana* on grapes in Italy (Marchesini and Monta 1994).

Given the ease of raising *E. steffani* on a substitute host (Redolfi and Campos 1998) and the fact that other species of *Elasmus* are being used for biological control (Ubandi et al. 1988; Bastian and Hart 1989; Kovalenkov et al. 1991), the aim of this work was to study, in detail, the biology of this parasitoid raised on *Ephestia kuehniella* (Zeller) (Lepidoptera: Pyralidae) and to identify ways in which to enhance its production.

This study will lead to improvement of the basic knowledge on this species and provide practical information for studies concerning the use of *E. steffani* in programs of integrated management of olive orchards in either inundative or inoculative releases against *P. oleae.*

## Materials and Methods

Populations of *E. steffani* and the substitute host *E. kuehniella* were obtained from laboratory-raised specimens. *E. kuehniella* was reared according to a method described by Celli et al. (1991), based on an insect artificial diet consisting of wheat germ and beer yeast (2:1), under laboratory conditions (20 ± 2° C, 60 ± 10% RH and 14:10 L:D). The last-stage larvae were chosen and wrapped in fine white paper, and afterward they were exposed to the *E. steffani* adults. In order to maintain the population size of *E. steffani* adults, 100 parasitoid couples (10 days old) and 300 *E. kuehniella* larvae were placed in wooden boxes (40 × 39 × 37 cm) with a glass cover. The larvae were renewed every 24 or 48 hours, and the adults fed on a honey and water mixture (2:1) (Redolfi and Campos 1998). This rearing was done under laboratory conditions (26 ± 2° C, 60 ± 10% RH and 14:10 L:D).

All trials of this study were done at 26° C, 60% RH, and photoperiod of 14:10 L:D. All host larvae in the trials were previously wrapped in fine white paper to simulate a cocoon (Redolfi and Campos 1998).

### Duration of the stages of development of *E. steffani*

Three 2-litre glass flasks were used and placed horizontally with a round filter paper covering the bottom and closed by a nylon mesh. In each flask, 100 *E. steffani* couples (male and female) were placed, and they were fed on a honey and water mixture (2:1) (Redolfi and Campos 1998).

After 10 days, 300 *E. kuehniella* in the last larval stage were exposed to the parasitoid for 24 h. The paralyzed host larvae with parasitoid eggs were placed individually in plastic Petri dishes of 8.5 cm in diameter and examined under a microscope every 8 h. Upon pupation, the parasitoid pupae were isolated individually in 30-ml glass tubes having cotton stoppers and were kept until adult emergence. Just after the emergence, the adults were separated on male-female pairs and placed in 30 ml tubes, where they were fed on a honey and water mixture (2:1).

### Pre-oviposition, oviposition and reproductive capacity of *E. steffani*

Ten pairs (female and male) of recently emerged *E. steffani* were placed individually in Petri dishes of 8.5 cm in diameter, and they were fed a honey-water mixture (2:1). They were exposed daily to five hosts in the last larval stage until the death of the female parasitoids. The days to oviposition, the number of eggs oviposited per day and per larva, and the number of larvae parasitized per day per female were recorded, and the means were determined. The parasitized larvae were isolated in Petri dishes.

### Mating and parasitism behavior in *E. steffani*

During the last two trials of this study, mating and parasitism behavior were observed, and various mating and ovipositing postures of the parasitoid, as well as time of behavioural events, were recorded.

### Parthenogenesis in *E. steffani*

A total of 10 recently emerged virgin female *E. steffani* were placed in a 2-litre glass flask and exposed daily to three host larvae. The parasitized larvae were isolated in Petri dishes until the emergence of the adults. The number of studied larvae was large enough to analyze the type of parasitoid parthenogenesis.

### Longevity and feeding of *E. steffani* adults

Each of 30 pairs of *E. steffani* adults, emerged within 24 h, were assigned to one of the three following sources of food: no food, a honey-water mixture (2:1), and a honey-water mixture (2:1) plus host larvae (3/d). Each pair of adults was placed in a glass tube stopped with cotton.

### Statistical analysis

The differences of duration of the developmental stages between males and females and the longevity of adults (males and females) in different feeding condition were tested for significance with the Kruskal-Wallis test (p < 0.01). Non-parametric tests were used because the data were not normally distributed, even after transformation.

## Results

### Duration of the developmental stages of *E. steffani*

No significant differences (p > 0.01) were found regarding the time required by the *E. steffani* males and females to complete their development from egg to adult. The duration under study conditions was approximately 11–15 days, with pupation occupying half of that time period (Table 1).

### Pre-oviposition, oviposition and reproductive capacity *E. steffani*

**Figure 1.**
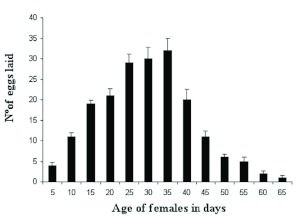
Oviposition rhythm of *Elasmus steffani* parasitizing *Ephestia kuehniella.* High quality figures are available online.

The mean pre-oviposition period was 8.9 ± 5.0 days, and the mean oviposition period was 30.4 ± 10.5 days. The mean reproductive capacity was 185.5 ± 62.3 eggs per female, with an average of 5.4 ± 0.9 eggs per day. The number of eggs per host larva was 4.4 ± 0.4, and the number of parasitized hosts per day averaged 1.3 ± 0.1 (Table 2). The oviposition rhythm observed (*n* = 10 females) reached its highest level at 35 days of age, with maximum oviposition between 15 and 40 days of age (Figure 1).

### Mating and parasitism behavior in *E. steffani*

Mating occurred both in the case of the females and males immediately after emergence. The male mounted the dorsal side of the female and made vibrating movements with its antennae, touching the antennae of the female and remaining in this position for an average of 17.4 ± 2.3 min. The copulation lasted for 2 to 5 s. In brood flasks containing 100 *E. steffani* pairs that had already mated, males were repeatedly seen on top of the females.

Regarding parasitic behaviour, upon detecting a host larva, the parasitoid would immediately insert its ovipositor into the host for 10 to 20 min, repeating this 4 or 5 times in zones near the head and behind the fourth pair of legs. During the insertion intervals, the parasitoid, using its legs and antennae to feel the larvae, remained stationary on the host for variable time periods. The process lasted between 2.15 and 2.34 h. When the host was immobilized, the parasitoid oviposited eggs near the larvae, preferentially near the ends, but rarely on the cuticle of the larvae itself.

All values correspond to 10 pairs (female and male) of recently emerged *E. steffani* placed individually in Petri dishes.

### Parthenogenesis in *E. steffani*

The offspring of virgin females were all male, indicating that *E. steffani* was capable of arrhenotokous parthenogenesis being of the arrenotokia type, as mentioned by Clausen (1940) for other species of Elasmidae.

### Longevity and feeding of *E. steffani* adults

Adults that were supplied with an aqueous honey solution in the absence of hosts had significantly longer longevities (p < 0.01) than those that were similarly fed and had access to hosts, and those with no food. In the treatments that provided adults with food, female longevity was three-fold (p < 0.01) that of males. The lack of feeding negatively affected survival in females (2.3 ± 0.7 days) and in males (2.2 ± 0.7 days), with survival time varying between 1 and 3 days without significant differences between sexes (Table 3).

**Table 1.**
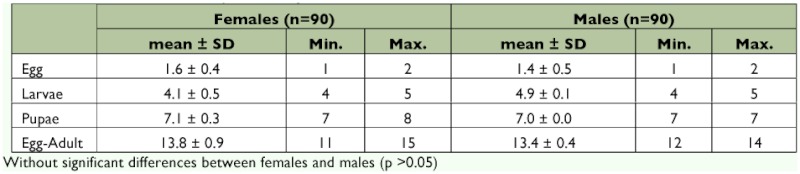
Duration of the developmental stages of *Elasmus steffani* at 26° C.

**Table 2.**
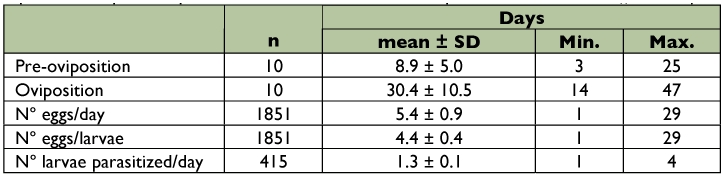
Pre-oviposition, oviposition periods and mean incidences of oviposition of *Elasmus steffani* on *Ephestia kuehniella.*

**Table 3.**
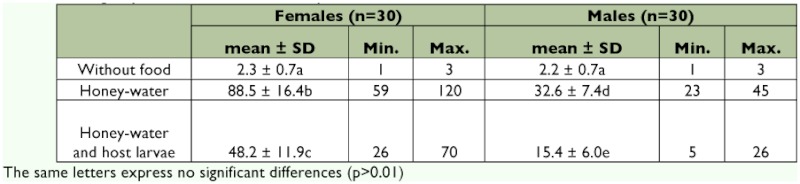
Longevity of *Elasmus steffani* adults provided with three different sources of food and no food.

A daily feeding rhythm was perceived in hours of light, principally 8:00 and 18:00 h, with peak activity between 9:00 and 11:00 h. Generally the females spent more time feeding (16.3 ± 4.6 min, *n* = 10) than the males (7.4 ± 1.8 min, *n* = 10).

Female *E. steffani* host fed upon *E. kuehniella.* For this, the parasitoid paralyzed the host larva and afterward, for 20 min sucked the haemolymph directly from the points where the ovipositor had been inserted. After feeding, the zone fed upon turned light-green in color and later black. Generally, the parasitoid did not oviposit in a host that had formerly been fed upon. On rare occasions, the male parasitoid also fed on the paralyzed host in places where the female had inserted the ovipositor, but only for a brief time (5 min). The female parasitoid only used 15% of paralyzed larvae to feed.

## Discussion

The lack of significant differences between males and females in terms of duration of development has been recorded also in the case of *Elasmus zehntneri* (Tanwar 1990). This could be due to the fact that the parasitoid is gregarious, and therefore the male generally does not need additional time to search for a female.

With regard to the duration of the larval and pupal stage of *E. steffani* (Table 1), Campos (1981) reported higher values (8.7 ± 0.3 and 11.7 ± 0.4 for larval and pupal stage, respectively), but it should be taken into account that the host was *P. oleae* and that the specimens were kept outside with daily variations in temperature of between 13 and 27° C. These fluctuations could prolong the duration of these developmental stages, as mentioned for *E. zehntneri* grown on *Tryporyza nivella intacta* Sn. (*Scirpophaga nivella*) (Ubandi et al. 1988; Tanwar 1990).

The duration of egg-to-adult development reinforces the observation by Clausen (1940) concerning the developmental period of Elasmidae, which requires 10 to 16 days in *Elasmus nephantidis* and an average of 14.5 days for *Elasmus hispidarum* at an average temperature of 29° C.

The pre-oviposition period for *E. steffani* is long and highly varied, which also has been mentioned for *E. zehntneri*, which averages 4.0 ± 1.8 days (range: 2–10) (Tanwar 1990).

This could be because *Elasmus* species, as the females of many synovigenic parasitoids, not only parasitize hosts but also feed on them to secure nutrients for the continued production of oocytes (Rosenheim and Rosen 1992; Jervis and Kidd 1986). The decision whether to host-feed or oviposit will depend on the number of mature eggs that a parasitoid is carrying, which is the reason why the status of the ovaries may determine the duration of any preoviposition period following eclosion (Jervis et al. 2005).

The mean number of eggs oviposited per female was greater for *E. steffani* than that recorded for other species of the genus *Elasmus.* That is, a range of 14 to 57 eggs was recorded for *E. nephantidis* (Ramanchandra-Rao and Cherian 1927), 69.37 and 53 eggs for *E. zehntneri* (Cherian and Israel 1937; Tanwar 1990), and 21–68 (42.70) eggs in 9 to 12 days for *E. brevicornis* (Peter and David 1990). In these cases, the shorter duration of the adult state of the parasitoids must have had an influence. In *E. steffani*, the number of eggs laid per female and per day was 4.4 ± 0.4 when the female had 4 or 5 host larvae. On the other hand, in the *E. zehntneri* case, the average number of oviposited eggs per larvae was 49.3 (Tanwar 1990). Since a host represents a limited amount of resource for gregarious parasitoids, clutch size is a variable feature of a species, because it is influenced by different factors such as host availability and size (Fellowes et al. 2007). Previous studies carried out on *E. steffani* showed that when the number of available hosts per female decreased daily, the clutch size increased, and at the same time, the superparasitism increased (Redolfi and Campos 1998). In contrast, when the host size was smaller, the clutch size was 2 ± 0.08 (Campos 1981).

The daily oviposition rhythm proved uniform in its rise and fall, with only one maximum peak at 35 days of age (Figure 1), without pronounced fluctuations, in comparison with other ectoparasitoid species (Redolfi et al. 1987).

The results indicate that in programs of biological control, depending on the food resources within the agricultural ecosystem, females should be released at 5 to 20 days of age.

The mating behaviour was similar to that described by De Bach (1985) for this same species and by Peter and David (1990) for *Elasmus brevicornis.* The only notable difference with respect to the latter species is that the *E. steffani* male remains for a longer time on top of the female and the female moves, transporting the male as she goes. In brood flasks containing 100 *E. steffani* pairs that had already mated, males were repeatedly seen on top of the females, behaviour that was never observed among isolated pairs. It is quite possible that this resulted from having a high number of specimens in a limited physical space. Studies of mating behaviour can help to identify the attributes that make a given species an effective biological control agent (Luck 1990).

Longevity, like fecundidty, is influenced by a range of physical and biotic factors such as temperature, host density, and food source (Jervis et al. 2005). The great effect that food availability exerted on the longevity of the adult of *E. steffani* indicates the need of a carbohydrate source for survival (Table 3).

Sugar consumption can increase the longevity and lifetime fecundity of many species of parasitic wasps. Consequently, for these insects the availability of sugar sources in the field is important for their reproductive success (Siekmann et al. 2001). In the case of unfed adults, the results do not coincide with those mentioned by Campos (1981), who established a greater longevity for males and females maintained without food, with significant differences between them. It is possible that the daily variations in the temperatures used by this author caused a longer duration, considering that at a constant high temperature, the parasitoid would more rapidly expend its energy reserves. Energy reserves, and consequently, life expectancy can decline at different rates depending on factors such as temperature and locomotory activities (Siekmann et al. 2001).

The function of host-feeding may be either to obtain energy or protein and other nutrients necessary for the production of eggs (Houston et al. 1992). Comsuption of host hemolymph improves longevity of some host-feeding wasp species but not in others (Jervis et al. 2005), and in the case of *E. steffani*, the longevity significantly diminishes when host larvae are supplied in addition to the honey-water mixture (Table 3). Why some species derive clear longevity benefits from host-feeding fluids whereas others do not is not clear, but it may have to do with interspecific differences in the nature of the nutrients consumed and with the amount of sugars present in the ingested fluids, in particular (Giron et al. 2002; Rivero and West 2005). In addition, the action of feeding on the previously paralyzed host larva could account for the presence in the field of paralyzed *P. oleae* larvae without eggs, as well as for similar observations by Peter and David (1990) on larvae of *Diaphania indica* paralyzed by *E. brevicornis.* Thus, in agreement with van Alphen and Jervis (1996), *E. steffani* appears to have a non-destructive feeding behaviour with regard to the host, which is characteristic of gregarious parasitoids in which the female oviposit fewer eggs (*n* = 1–2) or does not oviposit in larvae that have been paralyzed and used for food.

The greater longevity of *E. steffani* females compared with males under conditions of food availability has also been observed in *E. zehntneri* (Tanwar 1990) and *E. brevicornis* (Peter and David 1990), as well as in other species of parasitoids, which is reasonable in view of the copulating function of the male.
